# A Sustained-Release Membrane of Thiazolidinedione-8: Effect on Formation of a Candida/Bacteria Mixed Biofilm on Hydroxyapatite in a Continuous Flow Model

**DOI:** 10.1155/2017/3510124

**Published:** 2017-10-10

**Authors:** Mark Feldman, Julia Shenderovich, Eran Lavy, Michael Friedman, Doron Steinberg

**Affiliations:** ^1^Biofilm Research Laboratory, Institute of Dental Sciences, Faculty of Dental Medicine, The Hebrew University of Jerusalem, Jerusalem, Israel; ^2^The Institute for Drug Research, School of Pharmacy, The Hebrew University of Jerusalem, Jerusalem, Israel; ^3^Koret School of Veterinary Medicine, Robert H. Smith Faculty of Agriculture, Food and Environment, The Hebrew University of Jerusalem, Rehovot, Israel

## Abstract

Thiazolidinediones (TZDs) have been found to act as effective quorum sensing quenchers, capable of preventing biofilm formation. Our previous studies demonstrated a profound antibiofilm effect of the TZD derivative thiazolidinedione-8 (S-8), either in solution or incorporated into a sustained-release membrane (SRM-S-8) under batch conditions. In the present study, we used a constant depth film fermenter model in order to investigate the impact of SRM-S-8 on mixed* C. albicans*-*S. mutans* biofilm development, under flow conditions. We found that essential parameters of cospecies biofilm maintenance and maturation, such as metabolic activity, biofilm thickness, roughness, extracellular polysaccharides production, and morphology of both pathogens, were altered by SRM-S-8 in the flow system. We propose that prolonged and sustained release of S-8 in a flow-through system allows better penetration of the active agent to deeper layers of the mixed biofilm, thereby increasing its activity against both pathogens. In conclusion, the use of a locally applied sustained-release drug delivery system of S-8 can affect the dental polymicrobial biofilm, resulting in clinical improvements and a better patient compliance.

## 1. Introduction

The oral cavity is colonized by a plethora of microbial species, most of which can form biofilms that adhere to the dental oral surfaces.

The fungus* Candida albicans* is a human pathogen that is also found in the oral cavity. It is a dimorphic fungus that grows as both yeast and filamentous cells, and its local propagation leads to a fungal infection known as candidiasis. The oral clinical signs and symptoms of this infection include white patches on the soft tissue of the mouth, soreness, and problems with swallowing. Candidal biofilm formation is associated with extracellular polysaccharides (EPS) production and a morphologic switch to a pathogenic hyphal form [1].

*Streptococcus mutans* has long been recognized as one of the major causes of dental caries [2] due to its acidogenic and aciduric properties as well as its high biofilm formation efficiency.* S. mutans* biofilms progress when bacteria utilize dietary sugars and proliferate on tooth surface, forming microcolonies that are firmly imbedded within EPS [3]. EPS are synthesized by the bacterial extracellular enzymes glucosyltransferases (GTFs) and fructosyltransferases (FTFs) [4]. Moreover, EPS type glucans, synthesized by extracellular GTF, provide binding sites for bacterial colonization, thus promoting further development of the biofilms [5].

Cospecies communities of* C. albicans* and* S. mutans* are often found in the oral cavity.* C. albicans* has been found in close association with oral streptococci in denture- and mucosal-related diseases [6]. The coexistence of these species was demonstrated in early childhood caries [7] and also in deep carious lesions [8].* Streptococcus gordonii* and* Streptococcus oralis* form dual species biofilms with* C. albicans* on salivary glycoprotein-coated surfaces [9] or on epithelium [10]. Several species of oral streptococci promote growth, hyphae formation [11, 12], and virulence of* C. albicans* [13, 14]. Collaboration occurring in dual species biofilms of* S. mutans* and* C. albicans* leads to increased biofilm mass and cell densities [7, 15], thereby enhancing biofilm pathogenicity. It was shown that farnesol, a fungal quorum sensing (QS) signal, induces* S. mutans* biofilm cell growth, microcolony development, and GTF expression and activity [16]. The GTF-B enzyme exhibits high binding affinity to* C. albicans* [17, 18].

Oral infections such as candidiasis, caries, periodontal diseases, and root canal infections are of chronic nature; therefore prolonged therapy is required. However, most dental drugs are rapidly removed from the oral cavity; therefore, there is a need to develop sustained-release delivery systems which can prolong the time of residence of the drug at the active site [19]. Local sustained-release delivery systems increase the efficacy and the safety of the administered drug [20]. More so, due to the prolonged substantiality of the drug released from the sustained-release delivery system its effect also on the biofilm is more profound [21].

Sustained-release delivery systems, including varnishes and membranes intended for the oral cavity were able to maintain therapeutic concentrations of the active agent in the oral cavity for prolonged periods, along with negligible blood concentrations [22]. Several studies have demonstrated the* in vivo* effectiveness of antimicrobial or antifungal agents incorporated into sustained-release delivery systems, against* S. mutans* [23] or* C. albicans* [22, 24].

Thiazolidinediones (TZDs) have been documented as effective antibiofilm, quorum sensing quenchers [1, 25–28], which have the capacity to prevent biofilm formation* in vitro.* We found a profound antibiofilm effect of the TZD derivative, thiazolidinedione-8 (S-8), in solution [1, 29]. In order to enhance its biological activity, the TZD was incorporated into a sustained-release membrane (SRM) [28]. It was demonstrated that a SRM, containing the TZD derivative S-8 (SRM-S-8), inhibits fungal biofilm formation in a time-dependent manner, due to the prolonged release kinetics of S-8. Moreover, the TZD released from the membrane induced a significant decrease in the biofilm formed on the SRM-surrounding area. In addition, virulent parameters of matured biofilm such as attachment force, thickness, exopolysaccharide production, and morphogenesis of fungal cells were dramatically altered by SRM-S-8 [28]. It was also found that in its soluble form this agent alters the symbiotic relationship in the* C. albicans*-*S. mutans* dual species biofilm in a batch system [30].

For further investigating biofilm formation in the oral cavity, model systems are needed which provide constant flow conditions in combination with low shear forces. The constant depth film fermenter (CDFF) is widely used for the research of dental biofilm development [31–35]. The CDFF represents a flow system allowing for controlling the biofilm depth, flow of nutrients, and evacuation of metabolites.

In the present study we explored the antibiofilm effect of S-8, incorporated into sustained-release varnish membrane (SRM-S-8), on biofilm formation, using CDFF as a flow system and hydroxyapatite tablets (HA) as the surface.

## 2. Materials and Methods

### 2.1. Coating of the Hydroxyapatite Tablets

The SRM was prepared by dissolving polyethylene glycol (PEG-400, Sigma-Aldrich, Belgium), ammonio methacrylate copolymer type A NF (Eudragit® RL, Evonik Röhm GmbH, Germany), and S-8 (Daren Labs, Israel) in absolute ethanol (J. T. Baker, USA) until a homogenous solution was obtained [27]. The concentration of S-8 in the SRM was 20% of the dry weight. In order to determine the direct effect of SRM-S-8, one side of 9.5 mm diameter hydroxyapatite (HA) tablets (Clarkson Chromatography Products Inc., South Williamsport, PA, USA) was coated with the SRM preparation. The coated HA tablets were left to dry overnight at room temperature (RT). The tablets were weighed before and after coating, and the amount of S-8 was calculated as its weight percentage of the SRM-S-8.

### 2.2. Dissolution Studies

*In vitro* dissolution studies were performed at 37°C in a flow-through system using a syringe pump. Briefly, each HA tablet was inserted into a flow-through chamber (prepared in-house) of an approximate volume of 1.5 ml, attached to a 30 ml syringe filled with 1% (w/v) of sodium lauryl sulfate (Sigma-Aldrich) in water as the release medium. The syringes were then connected to a syringe pump (Harvard Apparatus 22, USA) that forced the liquid through the chamber and into a collecting tube at a rate of 1.25 ml/h. After 24 h, the syringes were refilled with 30 ml of the release medium, and the flow-through process continued for another 24 hours. The collecting tubes were replaced at designated time points, and S-8 concentrations in the samples were determined by HPLC. At the end of the experiment, each HA tablet was immersed in absolute ethanol to dissolve the remaining S-8, in order to determine the residual concentration that had not been released.

### 2.3. S-8 Analysis

The amount of S-8 released from the SRM-S-8 coated HA was determined by HPLC, using HP 1050 HPLC equipped with a UV detector. Separation was performed on a Hypersil Gold C-18 column (250 × 4.6 mm 5 *μ*m, Thermo Scientific, USA). The mobile phase was acetonitrile : water (70 : 30% v/v) and the flow rate was 1 ml/min. S-8 was detected at 280 nm. The calibration curve was linear at 1.5–100 *μ*g/ml range [27].

### 2.4. CDFF Flow System

**Figure 1:**
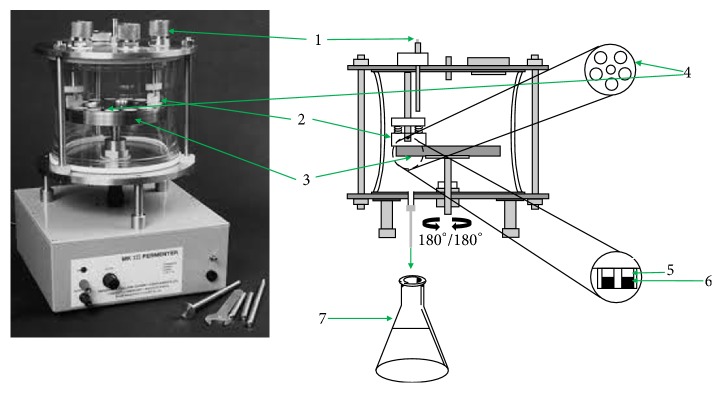
*CDFF flow system (Greenman et al., 2013; with few modifications made by authors)*. (1) Sample port × 3. (2) PTFE scraper bar × 2. (3) Turntable. (4) PTFE plug × 15. (5) Formed biofilm. (6) HA tablet-SRM (control/S-8). (7) Waste flask.

The controlled release film fermenter (CDFF) consists of a glass vessel which houses a stainless steel rotating disc, containing 15 PTFE plugs (18.0 mm diameter) [31, 32]. The HA tablets were placed onto the plugs. The surface level of the plugs can be controlled to ensure a constant depth of the biofilm formed on top of the HA tablets. As the turntable rotates, a PTFE scraper bar moves on the surface of the disc. The PTFE bar allows the incoming liquid to reach the biofilm and maintains the biofilms, once formed, at a constant predetermined depth (Figure 1). Two 2 L flasks, containing BHI medium, were connected through a peristaltic pump to the incoming sample ports of the glass vessel, while another 5 L flask was connected to the waste port (Figure 1).

The CDFF system was autoclaved at 121°C for 60 min before use. After autoclaving, SRM and SRM-S-8 coated HA tablets were aseptically placed on top of the PTFE plugs and adjusted to a depth of 100 *μ*m. Next, 1% of sucrose was added aseptically to the flask containing BHI which served as a supplemented medium, while another flask with BHI was inoculated with the microbes, as described below.

### 2.5. Dual Species Biofilm Development in CDFF

Precultures of* S. mutans* UA159 and* C. albicans* SC5314 were inoculated from several isolated colonies, grown separately for 18 h, in 5% CO_2_ at 37°C, and harvested by centrifugation (3136*g*, 10 min, 4°C). Next, both microbial cultures were diluted to OD_595_ = 0.1 in BHI medium and incubated overnight, in 5% CO_2_ at 37°C in 2 L BHI containing flask. After incubation the flask was connected to the CDFF as described below.

The assay was performed similarly as described previously [33]. Briefly, biofilms were allowed to develop in CDFF on SRM/SRM-S-8 coated HA tablets for 48 h, in a 5% CO_2_ atmosphere, at 37°C. The mixed inoculum and supplemented medium were pumped into the CDFF just in front of the PTFE scraper blade at a rate of 40 ml/h. The turntable rotated at a speed of 3 rpm and the fermenter was supplied with air, which was allowed to diffuse through sterile filters. The CDFF was operated in reciprocal mode (180° oscillation) (Figure 1). In order to prevent any contact between treated and untreated biofilms, SRM coated HA tablets were used as a placebo control on one side of the rotating disc, while SRM-S-8 coated HA tablets were inserted on the opposite side of the disc. Therefore, the liquids removed by the scraper blade from control and treated samples did not interact. After biofilm formation, all HA tablets were removed aseptically from the CDFF, washed 3 times in sterile PBS, and processed for further analysis as described below.

### 2.6. Microbial Metabolic Activity of the Formed Biofilms

The assay measuring metabolic activity was performed as described previously [30], with several modifications. Briefly, the mixed biofilms of* C. albicans* and* S. mutans* formed on SRM/SRM-S-8 coated HA tablets in the CDFF were washed with sterile PBS. Next the biofilm was overlaid with 100 mM of 3-(4,5-dimethyl-2-thiazolyl)-2,5-diphenyl-2H-tetrazolium bromide (MTT) and incubated for 2 h at 37°C. Under these conditions, the lightly yellowish MTT is reduced to a blue tetrazolium salt accumulated within the metabolic active biofilms. The dye on the HA tablets was dissolved in DMSO and the absorbance value was measured at 570 nm. The accumulation of tetrazolium salt by the reduction of MTT is proportional to the number of metabolically active cells growing in biofilm. Three independent experiments were performed.

### 2.7. Morphology of Mixed Biofilm

The assay was performed similarly, as described previously [30]. After washing, the HA tablets were immersed in 4% formaldehyde for 1 h at RT. The bacteria and fungi in the mixed biofilms formed on SRM/SRM-S-8 coated HA tablets were then visualized using an analytical Quanta 200 Environmental High Resolution Scanning Electron Microscope (EHRSEM) (FEI, Eindhoven, The Netherlands) at 2,000x magnification. At least three random fields were observed and analyzed. Three independent experiments were performed.

### 2.8. Confocal Laser Scanning Microscopy (CLSM) of Mixed Biofilms

The assay was performed similarly, as described previously [7, 30]. Mixed inoculum was prepared as described above, but instead of* C. albicans* SC5314 wild type we used strain* C. albicans* SC5314 carrying the GFP reporter gene (*C. albicans*, GFP) [36], (green color) kindly provided by J. Berman (Tel Aviv University, Israel). In order to label* S. mutans* EPS, 1 mM of Alexa Fluor 555-labeled dextran conjugate (red color) (10,000 MW, Molecular Probes Inc., Eugene, OR, USA) was added to the medium prior to biofilm formation, as described previously [30, 37]. Forty-eight-hour biofilms, developed on SRM/SRM-S-8 coated HA tablets, were washed with PBS and incubated for 45 min with concanavalin A-Alexa Fluor 647 conjugate (ConA; 25 mg/ml) (Invitrogen, Carlsbad, CA, USA). Con A (excitation wavelength 650 nm and emission at 668 nm) (yellow color) selectively binds to the glucose and mannose residues of fungal cell wall exopolysaccharides (EPS) [30, 38]. Finally, streptococcal cells in cospecies biofilms were labeled by an immunofluorescence method developed in our laboratory and published in Abcam Review (2016, July). Briefly, after fungal EPS staining, biofilms were washed with PBS and fixed in 4% formaldehyde for 1 h at RT. Next, they were incubated for 1 h in PBS containing 1% bovine serum albumin (BSA) and then with rabbit anti-*Streptococcus mutans* polyclonal antibody (1 : 500; Abcam, Cambridge, UK) in PBS-1% BSA (1.5 h) followed by Alexa Fluor 405-conjugated goat anti-rabbit IgG H&L antibody (blue color) (1 : 500; Abcam) in PBS-1% BSA (1 h). Stained EPS and microorganisms were observed with a Zeiss LSM 510 CLS microscope (Carl Zeiss, Oberkochen, Germany). Three-dimensional images of the microbes and EPS distribution within biofilms were constructed using Zen2009 software (Carl Zeiss). At least three random fields were observed and analyzed, and three independent experiments were performed. The amount of each microbial species as well as individual EPS production by* S. mutans* and* C. albicans* in each sample was calculated as a color-appropriated fluorescence intensity, using ImageJ v3.91 software (http://rsb.info.nih.gov/ij). The data are presented as amount of fungal and bacterial cells as well as individual EPS production by* C. albicans* and* S. mutans* cells in each layer of the biofilm (5 *μ*m). The percentage of total EPS production and total biomass in mixed biofilms formed on SRM-S-8 coated HA tablets was calculated as area under the curve (AUC) and compared to control SRM coated HA tablets (100%).

### 2.9. Surface Roughness of Formed Biofilms

Surface roughness of formed biofilms was evaluated using a Veeco Dektak 150 profilometer (DektakXT, Bruker, Bruker Nano Surfaces Division, Tucson, AZ, USA) [39]. Surface roughness was determined as average roughness (Sa ) and root mean square roughness (Sq). The Sa and Sq parameters represent an overall measurement of the biofilm surface topography. Sa expresses an absolute value of the differences in height of each point compared to the arithmetical mean of the biofilm surface. Sq represents the root mean square value of height values within the biofilm area. The profilometer was operated as follows: a 2 *μ*m stylus was moved in contact with the tested specimen during 60 sec, with a 1 mg force, and on 1.078 mm from the center of the slab. Data were acquired and analyzed with the Vision 64® 5.40 software (Bruker Corp., USA).

### 2.10. Statistical Analysis

Means of three independent experiments were calculated. The statistical analysis was performed using Student's *t*-test with a significance level of *P* < 0.01 as compared to controls.

## 3. Results

### 3.1. Kinetics of S-8 Release from the SRM-S-8-Coated HA in a Flow-Through System

**Figure 2:**
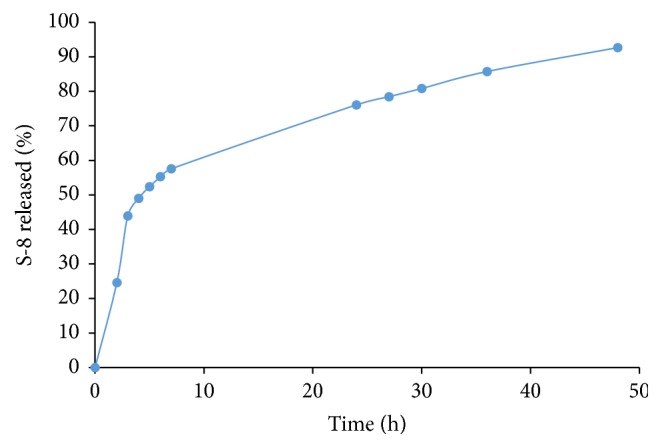
*S-8 release kinetics from SRM coated hydroxyapatite tablets in a flow-through system*. Release of S-8 is expressed as percentage of total S-8 dose, where 100 percent represents the amount of S-8 released in 48 hours plus the amount of residual unreleased S-8. Three independent experiments were performed.

The results shown in Figure 2 demonstrate the release of S-8 from the SRM coated HA tablets over a period of 48 h. A gradual release of the drug was detected during the initial 3 h. A total of 44% of the S-8 was released from the SRM within the initial 3 h. However, after this time period, the release rate of S-8 decreased, reaching 72% after 24 h. A total of 92% of the S-8 incorporated into the SRM coated HA tablets was released over the 48 h.

### 3.2. Metabolic Activity of Mixed Biofilm Cells

**Figure 3:**
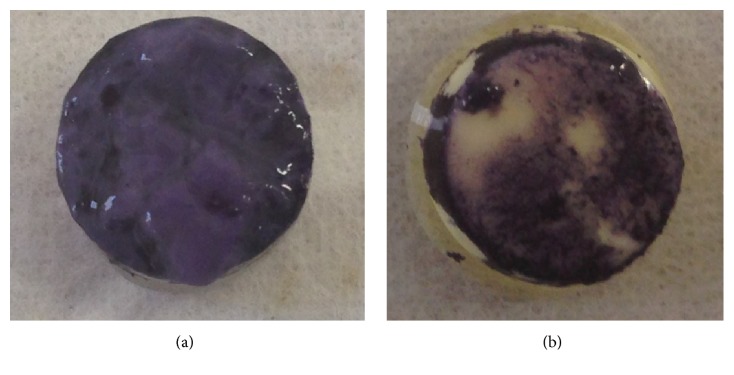
*Bacterial metabolic activity in the formed biofilms*. The MTT assay was used to determine the effect of SRM-S-8 on biofilm metabolic activity. Representative images are shown of (a) control biofilms formed on SRM coated HA and (b) treated biofilms formed on SRM-S-8 coated HA.

The MTT assay was conducted to determine the effect of SRM-S-8 on biofilm metabolic activity. A significant decrease in metabolic activity of biofilms formed on SRM-S-8 coated HA tablets was observed (Figure 3(b)) compared to control SRM biofilms (Figure 3(a)). The amount of metabolically active microbial cells was reduced by 65% (*P* < 0.01) in biofilms treated with SRM-S-8 (OD_595_ of 1 : 10 diluted sample = 0.16 +/− 0.078^*∗*^ (35%)) as compared to control (OD_595_ of 1 : 10 diluted sample = 0.45 +/− 0.68 (100%)).

### 3.3. Morphology of Mixed Biofilms

**Figure 4:**
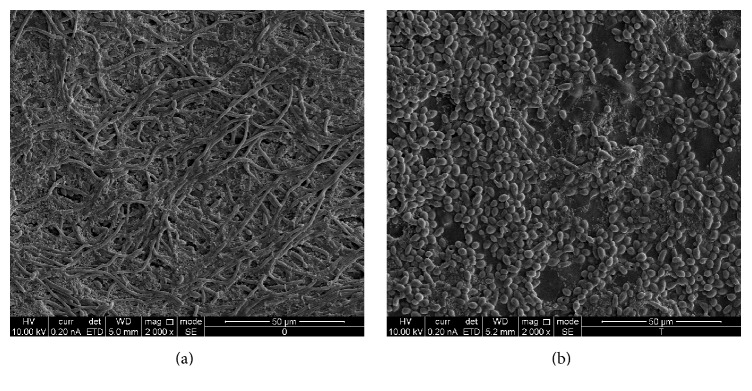
*Morphology of mixed biofilm*. The morphology of bacteria and fungi in the mixed biofilms formed on SRM coated HA (a) and SRM-S-8 coated HA tablets (b), visualized using Environmental High Resolution Scanning Electron Microscope at 2,000x magnification. At least three random fields were observed and analyzed, from three independent experiments.

SEM images showed that S-8 incorporated into SRM had a profound influence on the morphology and structure of mixed biofilms developed on the HA tablets. As shown in Figure 4, untreated control biofilm contained long matured filaments of* C. albicans* forming well-developed mycelium, while the streptococcal cells appeared in a form of numerous clusters attached to fungal hyphae cells (Figure 4(a)). In contrast, biofilms formed on HA tablets coated with SRM-S-8 consisted of candidal yeast form only, while bacteria were mostly in a single chain form, which spread sporadically within the dual species biofilm. Furthermore, in spite of the firm coaggregation with candida hyphae cells in control biofilm (Figure 4(a)), only a minority of* S. mutans* cells adhered to the yeast form of* C. albicans* in SRM-S-8 cospecies biofilms (Figure 4(b)). Moreover, the amount of* S. mutans* cells was obviously higher in untreated controls as compared to SRM-S-8-treated samples. Furthermore, an obvious decrease was observed in the density of biofilms developed on SRM-S-8 coated HA tablets as compared to control biofilms formed on SRM coated HA tablets.

### 3.4. Microbial and EPS Composition in Mixed Biofilms

**Figure 5:**
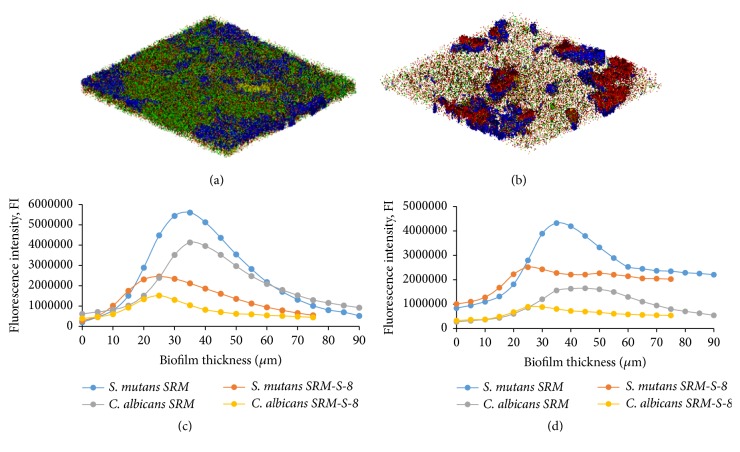
*Microbial and EPS composition in mixed biofilms*. CLSM merged images of biofilms of* C. albicans* and* S. mutans* microbial and EPS composition in control SRM coated HA (a) and treated SRM-S-8 coated HA tablets (b). Green color represents fungi and blue bacteria, yellow represents fungal EPS, and red represents bacteria. Magnification: 10x. At least three random fields were observed and analyzed, from three independent experiments. (c) Quantitative measurement of microbial composition. (d) Quantitative measurement of EPS production.

CLSM analysis demonstrated an alteration of biomass and EPS production in the mixed biofilm formed onto SRM-S-8 coated HA tablets as compared to control SRM coated ones. Total microbial volume and total EPS synthesis were reduced in the presence of SRM-S-8 by 59% and 37%, respectively, as compared to control (Table 1).* C. albicans* biomass (Figure 5(c)) and its EPS (Figure 5(d)) were reduced in the presence of SRM-S-8 by 68% and 47%, respectively, as compared to untreated control biomass and EPS (Table 1).* S. mutans* biomass (Figure 5(c)) and its EPS (Figure 5(d)) were decreased by 53% and 34%, respectively, in the SRM-S-8 treatments (Table 1) as compared to untreated control biomass. Moreover, as shown in Figures 5(c) and 5(d), total biofilm depth was reduced by 15 *μ*m (17%) (from 90 *μ*m in control to 75 *μ*m in treated sample) in the presence of SRM-S-8 coated HA tablets as compared with SRM coated HA tablets.

### 3.5. Profilometric Surface Analysis of Mixed Biofilms

**Figure 6:**
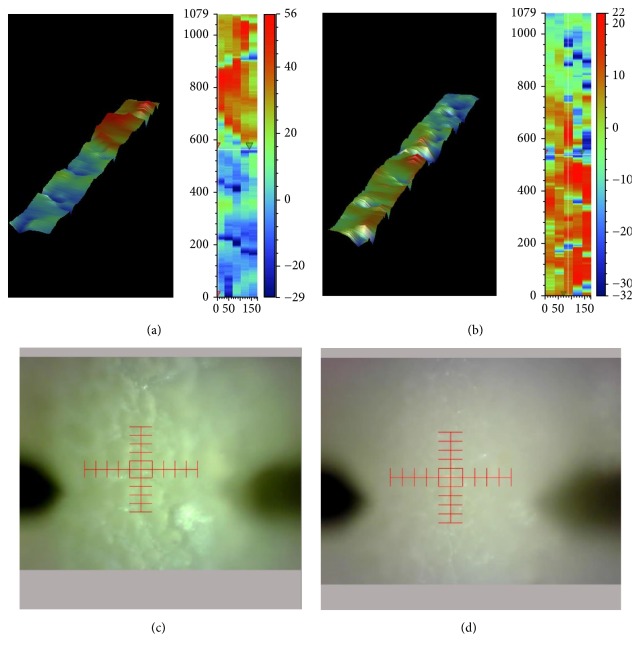
*Profilometric surface analysis of mixed biofilms*. 3D image of the biofilm surface topography of control SRM coated HA (a) and treated SRM-S-8 coated HA tablets (b). Biofilm images of control SRM coated HA (c) and treated SRM-S-8 coated HA (d). 10x magnification.

A significant difference (*P* < 0.01) was found between biofilms formed onto SRM-S-8 coated HA tablets and control SRM coated tablets as determined by the Sq and Sa values (Table 2). More so the surface of control biofilms showed high roughness (Figure 6(c)) characterized by deep pits and many grooves (Figure 6(a)). In contrast, biofilms treated with SRM-S-8 were of smoother surface (Figure 6(d)), with small pits and few grooves (Figure 6(b)).

## 4. Discussion

Since both microorganisms,* C. albicans* and* S. mutans*, exhibit virulence properties, it is of importance to control their presence in the oral cavity [40].

Our previous study demonstrated that, in its soluble state, S-8 reduced the total biomass of a cospecies* C. albicans*-*S. mutans* biofilm developed in a batch environment [30]. However, the bacteria/fungi ratio was modified in favor of streptococci with increasing concentrations of soluble S-8.

In this study we further examined the effect of S-8 now imbedded in a SRM coating HA surface and in a continuous flow system. In addition to the reduction of total biomass by SRM-S-8, each individual microbe biofilm formation was inhibited by this pharmaceutical formulation. Previously we found that* S. mutans* are less susceptible to the soluble form of S-8 than* C. albicans* [30]. We proposed that sustained release of S-8 allows better penetration of the active agent to the deeper layers of the mixed biofilm, thereby enhancing its activity against both pathogens. Indeed, it was documented that S-8 incorporated into the polymeric membrane is still active after 72 h of release. Furthermore, as previously reported [28], gradual release of S-8 from the SRM leads to a cumulative inhibitory effect and supports the prolonged activity of the formulation.

Our previous study [28] demonstrated the increased pharmaceutical potential of slow released S-8 against* C. albicans* monospecies biofilm. The virulence and structural characteristics of the* C. albicans* biofilm were affected by the sustained release of S-8 [28] in a batch experimental model. In the present study, we showed that essential parameters of cospecies biofilm maintenance and maturation, such as metabolic activity, biofilm thickness, roughness, EPS production, and morphology of both pathogens, were altered by SRM-S-8 in a flow system.

The ability of* C. albicans* and* S. mutans* to maintain their growth and metabolic activity when they are together may have clinical implications for the progression of infectious diseases. Numerous agents were shown to exhibit therapeutic potential against polymicrobial biofilms, via reduction of their metabolic activity [41, 42]. The decrease in metabolic activity of the mixed biofilm induced by SRM-S-8 found in the present work may indicate the high efficiency of this formulation against polymicrobial infections.

EPS production plays an essential role in interactions between* C. albicans* and* S. mutans* in regard to the modulation and development of virulent cospecies biofilms.* C. albicans* and* S. mutans* enhance binding to each other in the presence of sucrose [43, 44]. The CLSM results in our study showed reduction of total as well as each individual microbe's EPS produced in the mixed biofilm. This could be explained by the S-8′ delivery mode which mediated a decrease in total as well as single pathogen biomass. Furthermore, SEM images of biofilms formed on SRM-S-8 coated HA tablets showed a decrease of either bacterial or bacteria-fungi coaggregates indicating an alteration of EPS production. Lack of EPS limits the development of a mature biofilm and results in a thinner biofilm [45]. In accordance with these findings, we demonstrated that, with treatment with the SRM-S-8, biofilms were of thinner depth due to the EPS reduction.

The surface roughness of biofilms has been demonstrated to increase bacterial attachment to biofilms in continuous flow systems [46]. It was reported that biofilms which have a nonuniform, discontinuous, and greatly heterogeneous structure are characterized by high surface roughness [45]. In our study, untreated biofilm surface appeared as highly irregular and was characterized by deep pits and numerous grooves. This structural irregularity could be attributed to the presence of long candida filaments. In contrast, biofilms formed on SRM-S-8 coated HA tablets demonstrated more flat and smooth surfaces due to the presence of smaller yeast forms of* C. albicans*.

Dissolution studies performed under conditions of continuous flow have additional value when investigating delivery systems intended for the oral cavity. We have demonstrated [27] that under static experimental conditions the release of S-8 from SRM occurred via diffusion from a homogeneous matrix with a component of erosion. In the flow-through system, the small volume of liquid, to which the coated tablet is exposed at each time point, can cause momentary saturation conditions, which leads to a slower release than in a static dissolution system under sink conditions. Therefore, testing the release in the current tested flow system provides additional valuable information for future human applications of this device.

Those results, integrated with our previous study [27], demonstrated the ability to modify the SRM formulation in order to control the release rate. The present study indicates that a single application can affect the levels of the biofilm for at least 48 h. Future studies will be aimed at evaluating even longer-term effect of SRM-S-8 in flow-through conditions.

In conclusion, locally applied sustained-release drug delivery system can affect the dental polymicrobial biofilm, ultimately resulting in clinical improvements and a better patient compliance.

## Figures and Tables

**Table 1:** The percentage of the decrease of microbes and EPS in mixed biofilm composition in the presence of SRM-S-8 as compared to control SRM (100%).

Mixed biofilm composition	Percentage of decrease
Total EPS	37 +/− 3.48^*∗*^
Total microbes	59 +/− 2.75^*∗*^
*C. albicans*	68 +/− 4.1^*∗*^
*S. mutans*	53 +/− 1.9^*∗*^
EPS of *C. albicans*	47 +/− 2.7^*∗*^
EPS of *S. mutans*	34 +/− 5.7^*∗*^

*Note*. ^*∗*^Significantly lower than the value for the untreated control *P* < 0.01.

**Table 2:** Surface roughness parameters of mixed biofilms formed on SRM coated HA or SRM-S-8 coated HA tablets.

Sample	Sq, *μ*m		Sa, *μ*m	
	Mean	SD	Mean	SD
SRM	11.133	0.170969	9.037	0.965485
SRM-S-8	7.862	0.507185^*∗*^	5.798	0.866531^*∗*^

*Note*. SD = standard deviation. ^*∗*^Significantly lower than the value for the untreated control *P* < 0.01.
